# Protective factors against suicide among young-old Chinese outpatients

**DOI:** 10.1186/1471-2458-14-372

**Published:** 2014-04-16

**Authors:** Ying-Jen Chen, Yun-Fang Tsai, Shwu-Hua Lee, Hsiu-Lan Lee

**Affiliations:** 1 Division of Internal Medicine, Department of Internal Medicine, Chang Gung Memorial Hospital at Linkou, Tao-Yuan, Taiwan; 2 School of Nursing, College of Medicine, Chang Gung University, 259, Wen-Hwa 1st Road, Kwei-Shan, Tao-Yuan 333, Taiwan; 3 Department of Nursing, Chang Gung Memorial Hospital at Keelung, Keelung, Taiwan; 4 Department of Psychiatry, Chang Gung Memorial Hospital at Linkou, Tao-Yuan, Taiwan; 5 College of Medicine, Chang Gung University, Tao-Yuan, Taiwan; 6 Department of Nursing, Chang Gung Memorial Hospital at Linkou, Tao-Yuan, Taiwan

**Keywords:** Elderly suicide, Outpatients, Young-old people

## Abstract

**Background:**

Suicide is common among the elderly worldwide. However, no literature could be found on the beliefs/expectations that protect young-old people from attempting suicide. The purpose of this study was to explore young-old outpatients’ reasons for not killing themselves in Taiwan.

**Method:**

Data for this qualitative descriptive study were extracted from a large research series. From the 83 elderly outpatients in the original sample, 31 were chosen for this study because they were young-old (65–74 years old) and from two randomly selected medical centers in northern Taiwan. Data on participants’ reasons for not killing themselves in unhappy situations were collected in individual interviews using a semi-structured guide and analyzed by content analysis.

**Results:**

Analysis of interview data identified six major themes: satisfied with one’s life, suicide cannot resolve problems, fear of humiliating one’s children, religious beliefs, never thought about suicide, and living in harmony with nature.

**Conclusion:**

These identified protective factors (reasons for living) could be added to suicide-prevention programs for the elderly. Our findings may also serve as a reference for geriatric researchers in western countries with increasing numbers of elderly ethnic minority immigrants.

## Background

Suicide is common among the elderly worldwide [1]. Moreover, the suicide mortality rate of older Taiwanese was 35.8 per 100,000 in 2010 [2], higher than rates in the US, Germany, the UK, and Italy (6–24.3 per 100,000) [3]. With the increasing number of older people worldwide [1], suicide in this age group has become an important healthcare issue. Therefore, healthcare providers must be aware of this issue to prevent older people from completing suicide.

Indeed, two-thirds or more of older people who died by suicide in the US were seen by primary care physicians within a month of their death, and up to one-half within 1 week [4,5]. Similarly, 72.6% of people in a national health insurance database who completed suicide in Taiwan were seen by a physician within 1 month of their death [6]. These data highlight the importance of executing suicide prevention in outpatient settings.

Among the elderly (≥65 years old) in developed countries, the young-old (65 to 75 years old) are in transit from the workplace to retirement [7,8]. Retirement was found to be a life stressor for older people living in western countries due to reduced income, loss of role or work identity, and a smaller social network [9,10]. Many retired older people may need to care for grandchildren or ill family members [7,8] and face rearrangements in their lifestyle. However, some studies have found that retirement has a positive impact by relieving retirees’ job strain [11] as well as reducing headaches [11], mental and physical fatigue, and depressive symptoms [12]. Conversely, retirement is commonly viewed in Taiwanese society as connected to aging, loss of productive ability, and becoming less motivated to achieve [13]. This stigma has led to retirement becoming associated with devaluing one’s existence [13]. Whether retirement leads to negative or positive outcomes, it is accompanied by many changes that require some adjustment abilities. Some young-old people view retirement as an opportunity and develop ways to adapt, whereas others may have difficulty adjusting, which could lead to depression and suicidal ideas or even suicidal behaviors [14].

Suicide is influenced by interactions among biological, genetic, psychological, social, environmental, and situational factors [15]. Among these factors, the most prevalent risk factor for elderly suicide is depression [16]. Therefore, depression screening and treatment is commonly suggested for suicide prevention among older people [17]. Thus far, most suicidal prevention studies for older people have been conducted in primary care settings [18]. For example, suicide ideation was reduced for up to 2 years among older US adults using collaborative care models in which depression treatment was augmented in primary care with expert mental health consultation and medication recommendations, psychoeducation, and the option of brief psychotherapy [19,20]. However, reducing the frequency or severity of thoughts about suicide does not necessarily indicate reduced suicide risk [17]. Moreover, older people are not easily encouraged to voluntarily undergo depression screening in clinical settings. This reluctance is due to the stigmatization of mental disorders in Western culture, but can be more severe in Asian culture where mental illness is perceived as humiliating for the entire family [21]. Since stigmatization is a barrier to older people seeking depression screening and treatment, they may not respond to recommendations for such screening and treatment.

In suicide research, a person’s beliefs and hopeless expectancies are related to whether one attempts or completes suicide [22]. Since the beliefs and expectations of non-suicidal young-old people about suicidal behaviors may be considered protective factors against suicide, they are important to understand because this population is in transit from the workplace to retirement, which has special cultural meaning in Taiwan. Moreover, targeting this sub-population facilitates developing a model of suicide prevention in all older Taiwanese outpatients. Understanding these factors can help healthcare providers prevent suicide among the elderly not only by advocating depression screening and treatment, but also by enhancing protective factors (beliefs and expectations). Some protective factors against suicide identified in older people include sense of belonging [23,24], reasons for living [23], problem- and emotion-focused coping [25], and perceived social support [26]. However, no literature could be found on the beliefs/expectations that protect young-old people from attempting suicide. Moreover, few studies have addressed the topic of suicide in older non-psychiatric outpatients at general hospitals. To address these gaps in the literature, this study aimed to explore the reasons for young-old outpatients not killing themselves in Taiwan.

## Method

### Design

This qualitative descriptive study was part of a large research series to develop a suicide-prevention model for older people. Related findings have been published [27]. Data were collected by individual interviews from 2011 to 2012. In reporting this qualitative research, we have adhered to the BMC RATS guidelines.

### Sample and setting

Data for this study were extracted from a large research series. The original sample of elderly outpatients was recruited by convenience from three randomly selected medical centers, two in northern Taiwan and one in southern Taiwan. Participants were included if they met these criteria: 1) ≥65 years old, 2) without severe cognitive deficit (Chinese version Mini-Mental State Examination [MMSE] score ≥16 for those without formal education; MMSE score ≥20 for primary school graduates or above [28]), 3) they were outpatients in internal medicine clinics at the selected hospitals, and 4) self-reported never having suicidal ideas. Elderly outpatients were referred by their clinical physicians since they are responsible for documenting their patients’ health information. In our original research proposal, we considered age a possible factor influencing older peoples’ suicidal behaviors and protective factors. Therefore, during data analysis we divided data by participants’ age into three groups: young-old (65 to 74 years), old (75 to 84 years), and old-old (≥85 years). Of 83 transcripts from elderly outpatient participants in the original sample [27], 31 were chosen because participants were outpatients in the two randomly selected northern hospitals and were 65 to 74 years old.

### Data collection

Data were collected by a trained research assistant (RA, see training details below) in 31 audiotaped individual interviews, each lasting 20 to 60 minutes. Interviews were conducted in Mandarin Chinese in a private room at the selected hospitals. The RA encouraged participants to share their experiences by using a semi-structured interview guideline (e.g., “Have you heard of elderly suicide before?” “People commonly encounter some frustration or unhappy things in life. What are your reasons for not using suicide to deal with unhappy situations in life?”). In this series of studies, reasons for not executing suicide were considered protective factors against suicide. Participants were also told that suicide is the act of deliberately killing oneself [29] and suicidal behavior refers to attempting or completing suicide. Immediately after each interview, the RA used memos and a reflective journal to record observations about participants’ behavior during interviews and ideas about coding, respectively. Data collection and analysis were concurrent, with data collected until analysis showed no new themes (data saturation) by age group (young-old, old, and old-old) and participating hospital.

The RA was an experienced nurse who had been working on our research team’s suicidal studies more than 5 years. For this study, she attended at least three training sessions. In the first session, the second author explained the study purpose, design, and content to the RA. In the second session, the RA practiced using the interview guide with two patients until she became familiar with it. In the third session, the RA practiced observing non-verbal behaviors and taking notes by observing some patients during the practice interview.

### Ethical considerations

After the Chang Gung Memorial Hospital’s institutional review board approved the study, the RA approached elderly outpatients referred by physicians. The RA described the study purpose and procedure to outpatients, the required time commitment, confidentiality, and their right not to participate or to withdraw from the study at any time, and obtained their written consent to participate. Elderly outpatients received no compensation for their participation.

### Data analysis

All audiotapes were transcribed verbatim (in Mandarin) as soon as interviews ended. Transcripts were first compared with audiotapes for accuracy, and relevant information such as emotional content and nonverbal behavior was noted from memos and the reflective journal. All interview transcripts were then analyzed by content analysis to reveal themes in the interview data, as outlined below [30].

Transcripts were first analyzed individually with regard to memos and the reflective journal to identify key points. Next, all key points were listed and clustered into groups to form initial categories, which were used to recode transcripts. Categories across all transcripts were then listed and clustered into groups based on similarity and overlap. This grouping was refined to initially identify main themes. Coding and analysis of data from transcripts, memos, and the reflective journal continued iteratively until no new themes emerged. Finally, we labeled themes using participants’ own words and selected representative quotations. Member checking was then used by inviting two participants to review and check the authenticity of our findings. After they affirmed our analysis, the second author translated the Mandarin themes into English. The equivalence of the Mandarin and English themes was validated by comparison and discussion among the authors and a bilingual (Mandarin/English) expert in qualitative research.

### Rigor

Trustworthiness of the data was established by first three authors’ prolonged engagement with transcripts, methodological triangulation, participant observation, peer debriefing, and reflective journaling [31]. Method triangulation involved comparing data from transcripts with data from other sources (memos, reflective journal, and debriefing notes). For peer debriefing, all authors discussed the analysis with experts in qualitative research. To ensure confirmability, the second author made memos and kept a reflective journal on the decisions made throughout the study, thus allowing audit. Finally, reflexivity was promoted by all authors examining their own positions, beliefs, and values and their effects on the research. These effects were minimized by the second author’s reflexive journal and discussion among all authors.

## Results

The 31 participating young-old people were on average 70.3 years old (SD = 2.71, range = 65–74). The majority was female (58.1%), had graduated from a primary school or below (58.1%), and was married (58.3%). For details, see Table 1. In addition, most (74.2%) participants sought outpatient treatment at the clinics for hypertension, diabetes, or hyperlipidemia. The rest were treated for respiratory (16.1%), heart (6.6%) or gastrointestinal (3.2%) illness. Most (87.1%) of these young-old people had heard of elderly suicide. Their main information resources were watching television news (74.1%) and friends’ experiences (18.5%).

**Table 1:** **Demographic characteristics of young-old outpatients (*N*** **= 31)**

**Characteristic**	***n***	**%**	**Mean**	**SD**
Age (years)			70.3	2.71
Gender				
Male	13	41.9		
Female	18	58.1		
Formal education				
None	3	9.7		
Primary school	15	48.4		
≥ Junior high school	13	41.9		
Marital status				
Married	23	74.2		
Widow/widower	8	25.8		
Religious beliefs				
General folk beliefs	13	41.9		
Buddhism	12	38.7		
Other	6	19.4		
Living status				
Living with spouse	13	41.9		
Living with spouse and children	10	32.3		
Living with children	4	12.9		
Living alone	4	12.9		

Analysis of interview data indicated that participants’ reasons for not killing themselves were related to six themes: satisfied with one’s life, suicide cannot resolve problems, fear of humiliating one’s children, religious beliefs, never thought about suicide, and living in harmony with nature. Because chronic illness is a risk factor for suicide, participants’ illnesses and their protective factors were carefully reviewed for patterns of association. However, none was identified. Therefore, our findings could not be categorized by illness and represent participants’ pooled data.

### Satisfied with one’s life

Participants expressed satisfaction with their life. As C14 said, “My children are filial. We don’t have any financial pressure. We also have a nice neighborhood and no conflicts with other. My life is smooth.” Similarly, C69 said, “I don’t have any regrets in my life. My children are filial. I am not rich, but I don’t need to borrow money from others. Sometimes, I even can give my children money when they need help.”

Some participants described past experiences that impacted on their current life. As C31 said, “When I was young, I worked very hard to earn money. I had five children to raise. My wife’s health condition was poor, so she needed to visit doctors very frequently. I went through a very difficult time. Now I am old. My children can take care of us. Even though my knees used to hurt on rainy days, it is an old problem. I don’t need to worry about not having food to eat, clothes to wear, or money to spend. Why would I kill myself?” Similarly, C32 said, “Why would I do such a stupid thing? I worked very hard when I was young. Now, I am old and don’t need to worry about food and clothes. I also have money to spend. Why would I kill myself?”

### Suicide cannot resolve problems

Participants believed that everything can be resolved. As C55 said, “There is nothing that can’t be resolved. It takes time to overcome your problems. You need to be patient. Death cannot resolve any problems.” Similarly, C69 said, “Suicide is the last step. Nothing can be done after it.”

Other participants viewed suicide as an avoidant behavior when a person cannot deal with problems. As C83 said, “Suicide is a way to escape responsibility, but it cannot resolve problems.” Similarly, C61 said, “Life is full of responsibilities and you need to fulfill them. Suicide cannot resolve your problems. You cannot escape your responsibilities.”

### Fear of humiliating one’s children

Participants perceived that suicide would have negative effects on their children, who might be blamed for not showing filial respect for their parents and thus lose face. As C53 said, “People would ask, ‘Why did your father kill himself?’ There must be a reason. They might suspect that my son was not filial. That would increase his distress and humiliate him.” C53, C61 also said, “I felt suicide would make my children lose face. People would judge my children. They then would be stigmatized.”

### Religious beliefs

Some participants, mostly females, expressed a belief in reincarnation or transmigration. As C18 said, “Buddhism teaches transmigration. If you have suicidal behaviors in this life, you will carry it to your next life. It [suicidal behavior] will go with you forever. It is so painful. I don’t want to go through it.” Similarly, C55 said, “Suicide will turn up again in your next life and bring bad karma. I shall not kill myself.”

Other participants relied on religious beliefs as a way to attain peace and stability. As C58 said, “When I have problems, I always pray to Buddha. I then will receive the strength to deal with my problems. I don’t feel lost anymore. I also pray that my whole family will be at peace and healthy. I can rely on God.” Similarly, C77 said, “I chant twice a day, in the morning and at night time. I also go to a temple frequently. In this way, I receive the strength and support to live.”

### Never thought about suicide

Some participants reported they had never thought about suicide. C15, C82 said, “I don’t know the reason. However, I never had this thought [suicide] before.” Similarly, C20 said, “I just have never thought about suicide.”

### Living in harmony with nature

Participants expressed their philosophy of life as a way to cope with life’s challenges. As C16 said, “Life naturally goes through a process of birth, growing old, experiencing illness, and death. If you are sick, you die early. It is a law of nature. People should not fight against it [nature].” Similarly, C69 said, “Suicide is an unnatural way to die. People should die a natural death.”

## Discussion

This study contributes to suicide studies in the elderly by describing young-old people’s reasons for not killing themselves, which can be considered protective factors against suicide. When the young-old people in this study faced difficulties in life, they did not attempt or consider suicide for reasons related to six themes: satisfied with one’s life, suicide cannot resolve problems, fear of humiliating one’s children, religious beliefs, never thought about suicide, and living in harmony with nature. For these participants, satisfaction with one’s life was not related to the absence of illness, but to their adult children’s filial behavior, having enough money, and maintaining good relationships with others. Our participants’ view of illness as not a major concern for life satisfaction likely reflects their acceptance of and living with illness, even chronic illnesses, which are common among older people [32]. This acceptance was an important adaptive mechanism for these older people.

Conversely, children’s filial behavior is one of the most significant expectations for all Chinese parents [33,34]. As an old Chinese proverb says, “The purpose of raising children is to take care of you in your old age.” Therefore, Chinese adults are expected to care for their old parents. However, more families in today’s Chinese societies are nuclear rather than extended or multi-generational [33]. Indeed, the greatest proportion (41.9%) of our participants lived with their spouses only. Furthermore, some of our participants’ expectations of their children’s filial behavior had been lowered from living with their children to receiving a call from them 3 or 4 days per week or having a family reunion once per month. These lowered expectations of filial behavior may have increased their life satisfaction.

Another key point for life satisfaction in old age was having enough money. Even though we did not ask our participants about their income, several of them mentioned in the interviews that they were living on their own savings. They were proud of themselves for not adding to their children’s burden. Some participants even provided financial support for their children. This ability to be financially independent may have given them a sense of control and usefulness, increasing their self-confidence and life satisfaction. Finally, having harmonious personal relationships is also important for Chinese people. Having no conflicts or disputes with others reflects harmony and wisdom, two important characteristics of Chinese culture [35].

Some participants tended to deal with issues in daily life by rational analysis, i.e., by directly addressing and resolving problems. Suicide was perceived as escaping responsibility and not resolving problems. Such attitudes prevented our participants from using or considering suicide to resolve their problems. This finding suggests that strengthening rational analysis skills could be a strategy for suicide prevention in some older people. In this regard, cognitive-behavioral therapy, which is commonly used in psychiatric settings to change patients’ unrealistic ideas and adjust behaviors [36], may be adopted to help young-old people.

A unique and culturally significant finding of our study was that participants described not killing themselves because they were afraid of humiliating their children. This finding differs from child-related concerns [37] or concerns about family or others [38] reported as a reason for living in Western studies. Child-related concerns about suicide reflected the wish not to harm one’s children, not wanting others to take care of young children, or wanting to watch them grow up [37]. Concerns about family/others were related to older adults’ wish not to hurt their family, considering that their family depended on and needed them, or wanting to see their grandchildren grow up [38]. However, our young-old participants were concerned that their adult children would be blamed for not showing filial behavior and would lose face. Again, showing filial piety is a key relationship between parents and children in the Chinese family [33,34]. As mentioned before, our participants tended to have lowered expectations about filial behavior, but they still felt a need to consider their children’s face or standing in the Chinese community when making a decision about suicide. Indeed, the family bond is powerful in all Chinese family members’ decisions.

Religious beliefs were another reason for these young-old people not killing themselves. As two participants mentioned, Buddhism teaches transmigration or reincarnation. If you have suicidal behaviors in this life, these behaviors will go with you in your future lives for eternity. To avoid endless pain and suffering, our participants chose not to kill themselves. Similarly, other Asian countries with a stronger religious identity were shown to have lower suicide rates, e.g., Thailand and the Philippines where Buddhism and Catholicism, respectively, are widely practiced [39]. Likewise, considering oneself a religious person tended to serve as a protective factor against attempted suicide among community residents of Brazil, Estonia, the Islamic Republic of Iran, and Sri Lanka [40]. In addition, religious beliefs provided some of our participants with positive energy in the form of strength and inner peace, which helped them to deal with problems in their daily life. Other participants mentioned living in harmony with nature as another reason for not attempting or considering suicide. Although living in harmony with nature is actually advocated in Taoism [41], our participants did not identify themselves as Taoist. Therefore, we did not categorize this attitude as a religious belief but created a new theme.

Finally, some of our participants could not provide any reasons for their non-suicidal thoughts or behaviors. All of these participants were female. Data for the other five themes did not differ by gender. The majority of these female participants had less than a junior high-school education, possibly limiting their ability to reflect on and analyze their motives and beliefs about not killing oneself. Similar findings have been reported for older Taiwanese veterans’ home residents’ reasons for living [42].

Our participants did not express other reasons for living mentioned in Western studies [37,38], such as fears of suicide and of social disapproval about themselves. Fear of suicide may not have been mentioned by our participants since most of them had negative emotional reactions or judgmental attitudes toward elderly suicide. In addition, social disapproval in Western studies was related to older people themselves (e.g., I am weak and selfish, or I did not have control over my life) [37,38]. However, our participants were concerned about suicide resulting in social disapproval of their children for not showing sufficient filial piety. It is unclear why our participants did not mention fear of social disapproval for themselves. Further studies are needed to explore the relationship between fear of social disapproval about oneself and suicide in this age group.

Our findings differ from a recent report that older (mean age = 81.7 years) institutionalized Taiwanese males’ motivation to live was due to five factors: family member support, friend support, hope for the future, fear of death, and self-acceptance [43]. These different findings can be explained by the different study samples. Our participants were both male and female, but the previous study [43] included only males. Males and females have been reported to have different suicide-protective factors [44]. Another difference is that all of our participants were younger (mean age = 70.3 years) and living in the community.

Older Chinese people who live in the community and in institutions differ in their living environment, health condition, social network and support, or expectations for life. It is not common for older adults in Taiwan to live in a nursing home unless their daily care presents considerable difficulty for their adult children [45], who are expected in Chinese society to take on the responsibility of caring for their aging parents. Moreover, older Taiwanese nursing home residents consider a nursing home as ‘a temporary home to nurture health’ with highly structured lifestyle, restricted activities, safety concerns and social interactions with others [46]. All of the above may contribute to the different findings of our study and the previous one [43].

Even though suicide is influenced by interactions among biological, genetic, psychological, social, environmental, and situational factors [15], suicide is largely preventable [47]. Given the complexity of factors influencing suicidal behavior, international agencies stress that effective suicide-prevention strategies should focus on interdisciplinary collaboration, multidisciplinary approaches, and continued evaluation and review of at-risk patients [47]. Depression screening and treatment is commonly suggested for suicide prevention among older people [17]. Since the stigma of mental health issues is a barrier to older people receiving depression screening and treatment, a suicide-prevention program may be more acceptable if it not only advocates depression screening and treatment but also enhances protective factors (beliefs and expectations). Further studies could use our findings as a reference for developing a suicide-prevention video that advocates depression screening and treatment for the elderly as well as enhancing identified protective factors. This video could be regularly broadcast over local or national television or in the waiting rooms of outpatient clinics. Older people who view their counterparts sharing their own reasons for living may be persuaded to not consider or complete suicide. Moreover, education groups could be held in outpatient clinics to discuss depression screening and treatment, as well as protective factors against suicide.

### Strengths and limitation

Suicide is common among older people worldwide. Given the stigma associated with mental illness, including depression, it is important to execute suicide prevention in outpatient settings. Since stigmatization is a barrier to older people seeking screening and treatment, they may be more likely to accept a suicide-prevention program that integrates depression screening and treatment with suicide-protective factors. This study contributes to the literature on suicide in young-old people, specifically older Chinese, by providing information on their reasons for not killing themselves. However, this study had one limitation. The sample was recruited by convenience from two randomly selected hospitals in northern Taiwan. Thus, participants’ opinions may not represent those of the young-old from other parts of Taiwan or a randomly chosen sample. This limitation may have been minimized by the similarity of our sample’s male/female ratio (1: 1.4) to national data on older people (1:1.1) in Taiwan [48]. Further studies may consider using a random sampling strategy.

## Conclusions

In this qualitative study, we identified six major themes related to young-old outpatients’ reasons for not killing themselves: satisfied with one’s life, suicide cannot resolve problems, fear of humiliating one’s children, religious beliefs, never thought about suicide, and living in harmony with nature. These identified protective factors (reasons for living) could be added to suicide-prevention programs for the elderly. Our findings may also serve as a reference for geriatric researchers and clinicians in Western countries with increasing numbers of elderly ethnic minority immigrants [49], including Chinese or other Asians.

## Competing interest

The authors declare that they have no competing interest.

## Authors’ contributions

YJC assisted with designing the study, collecting and analyzing the data, and writing the article. YFT designed the study, supervised the data collection, performed data analyses, and wrote the paper. SHL and HLL assisted with collecting and analyzing the data, and writing the article. All authors read and approved the final manuscript.

## Pre-publication history

The pre-publication history for this paper can be accessed here:

http://www.biomedcentral.com/1471-2458/14/372/prepub
